# Health Literacy and Health Information Technology Adoption: The Potential for a New Digital Divide

**DOI:** 10.2196/jmir.6349

**Published:** 2016-10-04

**Authors:** Michael Mackert, Amanda Mabry-Flynn, Sara Champlin, Erin E Donovan, Kathrynn Pounders

**Affiliations:** ^1^ Stan Richards School of Advertising and Public Relations Moody College of Communication The University of Texas at Austin Austin, TX United States; ^2^ Center for Health Communication Moody College of Communication The University of Texas at Austin Austin, TX United States; ^3^ UTHealth School of Public Health Houston, TX United States; ^4^ Charles H Sandage Department of Advertising College of Media University of Illinois Urbana-Champaign Champaign, IL United States; ^5^ Mayborn School of Journalism The University of North Texas Denton, TX United States; ^6^ Department of Communication Studies Moody College of Communication The University of Texas at Austin Austin, TX United States

**Keywords:** health literacy, personal health information, biomedical technology, medical informatics

## Abstract

**Background:**

Approximately one-half of American adults exhibit low health literacy and thus struggle to find and use health information. Low health literacy is associated with negative outcomes including overall poorer health. Health information technology (HIT) makes health information available directly to patients through electronic tools including patient portals, wearable technology, and mobile apps. The direct availability of this information to patients, however, may be complicated by misunderstanding of HIT privacy and information sharing.

**Objective:**

The purpose of this study was to determine whether health literacy is associated with patients’ use of four types of HIT tools: fitness and nutrition apps, activity trackers, and patient portals. Additionally, we sought to explore whether health literacy is associated with patients’ perceived ease of use and usefulness of these HIT tools, as well as patients’ perceptions of privacy offered by HIT tools and trust in government, media, technology companies, and health care. This study is the first wide-scale investigation of these interrelated concepts.

**Methods:**

Participants were 4974 American adults (n=2102, 42.26% male, n=3146, 63.25% white, average age 43.5, SD 16.7 years). Participants completed the Newest Vital Sign measure of health literacy and indicated their actual use of HIT tools, as well as the perceived ease of use and usefulness of these applications. Participants also answered questions regarding information privacy and institutional trust, as well as demographic items.

**Results:**

Cross-tabulation analysis indicated that adequate versus less than adequate health literacy was significantly associated with use of fitness apps (*P*=.02), nutrition apps (*P*<.001), activity trackers (*P*<.001), and patient portals (*P*<.001). Additionally, greater health literacy was significantly associated with greater perceived ease of use and perceived usefulness across all HIT tools after controlling for demographics. Regarding privacy perceptions of HIT and institutional trust, patients with greater health literacy often demonstrated decreased privacy perceptions for HIT tools including fitness apps (*P*<.001) and nutrition apps (*P*<.001). Health literacy was negatively associated with trust in government (*P*<.001), media (*P*<.001), and technology companies (*P*<.001). Interestingly, health literacy score was positively associated with trust in health care (*P*=.03).

**Conclusions:**

Patients with low health literacy were less likely to use HIT tools or perceive them as easy or useful, but they perceived information on HIT as private. Given the fast-paced evolution of technology, there is a pressing need to further the understanding of how health literacy is related to HIT app adoption and usage. This will ensure that all users receive the full health benefits from these technological advances, in a manner that protects health information privacy, and that users engage with organizations and providers they trust.

## Introduction

Health literacy—how people obtain, understand, use, and communicate about health information to make informed decisions [1]—is related to a host of poor health outcomes and increased health care system costs. With approximately one-half to one-third of US adults struggling with health information [2,3], from reading medication labels to following instructions from health care providers, the need for improved models of communicating clear and compelling health information is pressing.

eHealth (the practice of using the Internet and telecommunication technology to provide health communication and services) [4] presents a powerful tool for bringing health information to low health-literate audiences in ways that are easier to access. Indeed, populations in which low health literacy is more prevalent, such as households with low incomes and racial or ethnic minorities [2], are also found to be the most likely to own and rely on a smartphone to access the Internet [5]. Searching for health topics is a common activity among those with smartphones; a recent survey from the Pew Research Center suggested that 62% of individuals who own smartphones used their phone to acquire information about a health condition or topic [5]. In this study, we further examined the relationship between eHealth and health literacy by exploring an emerging concept, that of health information technology (HIT), which ranges from personalized fitness trackers to apps on smartphones, to patient portals for electronic health record (EHR) systems.

The rapid adoption of mobile phones and smartphones among populations who are more likely to have low health literacy presents a tremendous opportunity for improving access to health information and tools to improve health [6]. eHealth interventions developed specifically to meet the needs of lower health-literate users can be more broadly acceptable to health-literate users too [6,7]. Overall, creating effective eHealth interventions is an opportunity that could be easily missed, however, if designers of personal HIT apps do not keep in mind the needs and preferences of lower health-literate audiences. Hayrinen et al argue that, as HIT continues to evolve, the “needs and requirements of different users [should be] taken into account” [8]. Similarly, Bickmore and Paasche-Orlow argue that, if researchers work to reduce the barriers related to accessing and using this technology, HIT may “level the playing field” for patients of low health literacy [9]. By enabling this group to receive health information at the right time and place, patients’ understanding and use of this information will be facilitated [9]. Ensuring the broadest possible successful adoption of HIT will ensure a new “digital divide” does not emerge between more health-literate users who can benefit from personal HIT apps and less health-literate users who might struggle to use them to their full potential.

As new HIT tools have become much more widely available, health-oriented apps designed for patients have exploded in recent years. There are now thousands of health-related apps offered through Apple and Android phone services available to patients for a wide variety of health concerns, from management of chronic illness management, to sleep behavior monitors, physical activity and educational and training videos, and calorie counters. For example, app searches performed by Eng and Lee [10] uncovered 240 applicable results for the Android platform when searching for “diabetes” and close to 600 apps designed for use on an iPhone. Additionally, recent industry reports indicate that the use of fitness and nutrition apps continues to grow in popularity as Americans are increasingly willing to use mobile phone apps to help manage their health [11]. Many of these apps are relatively affordable and are compatible with a variety of devices including mobile phones, tablets, computers, and wearable technology. The growth in this market over the past 5 years suggests that HIT tools are now available to a wider demographic, one that spans patients’ abilities to manage health information.

Another recent development in technology designed for patients is the creation of EHRs and patient portals, through which patients can directly access their health information when connected to the Internet. With the passing of the Health Information Technology for Economic and Clinical Health (HITECH) Act in 2009, there has been notable growth in the number of nonfederal acute care hospitals becoming equipped with and using EHRs in the United States [12]. Between 2009 and 2014, the percentage of these hospitals adopting basic EHR grew from 12.2% to 75.5% [12]. In 2014, 34.4% of the EHRs adopted offered patients “comprehensive” information, including notes and orders from their provider and nurse, laboratory analyses and results, and support for taking medications appropriately (eg, guidelines, interaction information, and dosing) [12]. Patients, then, have a great deal of their personal health information at their fingertips and can monitor changes in their health through a patient portal. Additionally, EHRs enable patients to contact their provider with questions about information presented in the EHR and changes over time. There is limited research available regarding the factors that determine whether a patient will use a patient portal or EHR. However, in one study, the use of a personal health record was determined by patients’ perceived ease of use of the technology, as well as their belief in the advantages offered by the technology and their ability to test-drive and witness the functions of the EHR [13]. Among hospitals that have not yet adopted EHRs, an increasing number have indeed been able to become equipped for EHR technology [12], and thus the availability of this technology is projected to continue to expand. Furthermore, health care providers are likely motivated to adopt EHRs by incentives provided by the federal government and to avoid penalties [14,15]. More research is needed to better understand patients’ reception of this technology.

A review of the recent literature in this area suggests that evidence on patients’ perceptions and use of HIT tools is rather limited. Most of the research in the area of HIT has focused on health care providers’ perceptions of and experiences with these technologies and their benefits to patient care as a whole [16-18], yet even these studies were noted as limited [16]. However, it is the hope that HIT tools will “improve the quality of health care [and] prevent medical errors” for patients [19] as well. Governmental agencies note that, by providing patients with HIT tools, they put the patients in charge of their health care [20]. Additionally, this may facilitate the concept of a patient-centered medical home, which aims to bring together patients, their providers, and technology to develop a central place of communication and treatment [21]. This fundamentally changes the paradigm of patient care as it works to minimize previous barriers to patients having direct access to their personal health files and creates situations in which patients might feel empowered to track and manage their health.

However, providing patients with opportunities to engage with their health information directly over electronic sources also puts patients’ private information at risk. This could come in two forms. First, patients who perceive themselves as having a high ability to manage health information may unknowingly share information they do not intend to and unknowingly share personal information they would prefer to be private. On the other hand, some patients may be reluctant to admit struggles and ask for assistance with health information, and thus may not make full use of HIT or could make mistakes that may compromise their personal information.

Privacy and the protection of personal health information varies across HIT apps, something perhaps not known by all patients. For example, EHRs must abide by the Health Insurance Portability and Accountability Act (HIPAA)’s Privacy Rule, which stipulates specific “safeguards” and rules about how a patient’s health information is handled and disclosed through an electronic platform such as a patient portal. Because the Internet is available to everyone, these regulations help ensure that a patient’s health information will not be “leaked” or be available to others who do not share an agreement with a health organization (such as an insurance company). These policies were set forth to “elicit greater consumer confidence, trust, and participation in electronic health information exchange” by patients of all backgrounds [22]. These regulations have extended privacy coverage so that some businesses such as Google are indeed held responsible for maintaining privacy of patient health information [13]. These policies, however, are limited to only EHRs and health information managed by health systems. As such, they do not yet apply to other HIT tools such as the aforementioned health apps and fitness trackers.

These types of privacy policies may lead to a greater sense of trust in the companies or institutions associated with various types of HIT. Trust is often an important factor contributing to the adoption of new technologies [23-25]; however, such policies could be misleading to patients who struggle with low health literacy, who might assume that all HIT have similar patient privacy rules and regulations. The degree to which a patient exhibits trust in institutions that may develop various HIT, such as health care organizations, the government, information technology companies, and media outlets, may influence their likelihood of adopting HIT and could be associated with health literacy level.

The purpose of this study was to investigate how health literacy might be related to use of a variety of HIT apps. Further, it was intended to investigate how health literacy is related to two crucial elements associated with HIT usage: (1) understanding privacy issues related to HIT adoption and (2) trust in various stakeholders associated in various ways with growth in HIT. As such, 4 research questions guided this research. (1) Is health literacy associated with a patient’s use of various forms of HIT apps including fitness and nutrition apps, activity trackers, and patient portals? (2) Is health literacy associated with a patient’s perceived ease of use and usefulness of these HIT apps? (3) Is a patient’s health literacy associated with perceptions of privacy associated with HIT apps? (4) Is a patient’s health literacy associated with perceptions of trust in various institutions (government, media, technology companies, and health care)?

The remainder of this paper provides an overview of research methods and a report of study results. This is followed by a discussion of the implications of this investigation for future research, practice, and policy. HIT has tremendous potential to improve the health of users, and this study is a crucial step toward better understanding how health literacy is associated with HIT adoption and ensuring that users of all levels of health literacy can realize those benefits.

## Methods

### Procedure

We recruited participants from an invitation-only research panel. All were enrolled members of the panel and received an email notification of their qualification for the study and a link to an online survey. The study took approximately 20 minutes to complete and participants were compensated for their time. The online survey included items to assess health literacy, participants’ use and perceptions of four different types of HIT, and demographic information. The study protocol was approved by the relevant institutional review board.

### Measures

#### Health Literacy

To measure health literacy, participants completed the task-based Newest Vital Sign (NVS) measure of health literacy. This measure asks patients to read and answer 6 questions about a nutrition label [26]. Sample questions include “If you eat the entire container, how many calories will you eat?” and “Pretend that you are allergic to the following substances: penicillin, peanuts, latex gloves, and bee stings. Is it safe for you to eat this ice cream?” These questions require participants to use basic quantitative (eg, 250 calories × 4 servings) and qualitative (eg, the list of ingredients includes peanut oil, and therefore someone allergic to peanuts should not eat the ice cream) problem-solving skills. Patients are awarded 1 point for each correct answer they provide. As such, health literacy scores using this measure range from a total of 0 to 6, where a score <4 indicates a potential for low health literacy [26]. The NVS is a valid and reliable measure of health literacy and commonly used in studies on this topic [26-32].

#### HIT Use

Participants were asked if they had ever used four different types of HIT: fitness apps (eg, C25K, MapMyRun, FitStar Personal Trainer), nutrition apps (eg, MyFitnessPal, Weight Watchers), activity trackers (eg, Fitbit, BodyBug, a pedometer), and patient portals (eg, BlueAccess, myUHC).

#### HIT Perceptions

For each HIT, participants were asked to indicate their degree of agreement on a 7-point Likert scale (1 = strongly disagree to 7 = strongly agree) with a statement related to perceived ease of use (eg, “Learning to use a fitness app is easy for me.”) and usefulness (eg, “Using a nutrition app is beneficial to me.”). Perceived ease of use and perceived usefulness are core constructs of the technology acceptance model [33] and are helpful concepts for understanding participants’ adoption and use of HIT.

#### HIT Privacy

Perceptions of privacy were assessed for each HIT: fitness apps (Cronbach alpha=.763), nutrition apps (Cronbach alpha=.779), activity trackers (Cronbach alpha=.795), and patient portals (Cronbach alpha=.821). Participants were asked to indicate their agreement with 6 statements using a 7-point Likert scale (1 = strongly disagree to 7 = strongly agree). Sample items are “I am certain that all the information I reveal on nutrition apps remains under my control” and “I tell intimate, personal things about me to be stored in nutrition apps without hesitation” [34].

#### Trust

Perceptions of trust were examined for four different institutions: government (Cronbach alpha=.925), media (Cronbach alpha=.868), technology companies (Cronbach alpha=.885), and the health care system (Cronbach alpha=.824). Two items assessed trust in each institution. Participants were asked to indicate their agreement with statements using a 7-point Likert scale (1 = strongly disagree to 7 = strongly agree); sample items are “I feel assured the government does a good job making laws that protect people’s health information” and “I feel the media does a good job monitoring issues related to health information privacy.”

#### Demographics

We collected specific demographic information on sex, race/ethnicity, age, income, and whether the participant worked in health care.

## Results

### Participants

A total of 5151 American adults reflecting the demographic composition of the United States in terms of sex, age, race/ethnicity, and socioeconomic status participated in this study. After removing participants with missing data, we included a total of 4974 participants for analysis. Table 1 shows the demographic distribution of the sample. Overall, 15.96% (794/4974) of the sample exhibited low health literacy, by achieving a score of ≤3 on the NVS measure of health literacy. In the full sample, 27.64% (1375/4974) indicated having ever used a fitness app, 33.89% (1686/4974) had used a nutrition app, 33.39% (1661/4974) had used an activity tracker, and 41.95% (2087/4974) had used a patient portal.

### Research Question 1

Research question 1 explored how the use of various HIT tools may differ between participants with adequate health literacy (NVS score ≥4) and those with less than adequate health literacy (NVS score ≤3) [26]. Cross-tabulation analysis indicated that adequate versus less than adequate health literacy was significantly associated with use of fitness apps, (χ^2^_1, N=__4974_=5.663, *P*=.02), nutrition apps (χ^2^_1, N=__4974_=18.885, *P*<.001), activity trackers (χ^2^_1, N=__4974_=54.754, *P*<.001), and patient portals (χ^2^_1, N=__4974_=102.642, *P*<.001). Across all HIT tools, fewer participants with less than adequate health literacy indicated technology use than those with adequate health literacy (Table 2).

### Research Question 2

Research question 2 further examined participants’ perceptions of various HIT; hierarchical linear regression analysis explored the association between perceived ease of use and usefulness for each technology and total NVS score. Specifically, we conducted eight regression models in which we regressed demographics (step 1) and total NVS score (step 2) onto perceived ease of use and perceived usefulness for four types of HIT (fitness apps, nutrition apps, activity trackers, and patient portals).

Overall, all eight models were significant (Table 3,Table 4,Table 5,Table 6), accounting for between 3.3% and 9.1% of total variance. Of most relevance to our study, NVS score was significantly associated with perceived ease of use and perceived usefulness across all HIT after controlling for demographics (see Table 3,Table 4,Table 5,Table 6 for demographic details).

**Table 1:** Participant demographics.

Characteristic		Mean (SD) or n (%)
Age in years, mean (SD)		16.7 (43.5)
Work in health care, n (%)		603 (12.1)
Male, n (%)		2102 (42.3)
**Race, n (%)**		
	White	3146 (63.2)
	Hispanic	671 (13.5)
	African American	794 (16.0)
	Asian	218 (4.4)
	Other	121 (2.4)
2-Year college degree or higher, n (%)		2980 (59.9)
**Household income in US $, n (%)**		
	<10,000	230 (4.6)
	$10,000–49,999	1908 (38.3)
	$50,000–99,000	1764 (35.5)
	≥$100,000	1068 (21.5)

**Table 2:** Health literacy × health information technology (HIT) use cross-tabulation (N=4974).

HIT	Health literacy	Used HIT, n (%)		χ^2^_1_	*P* value
		Yes	No		
**Fitness apps**				5.663	.02
	Low	192 (24.2)	602 (75.8)		
	Adequate	1183 (28.3)	2997 (71.7)		
**Nutrition apps**				18.885	<.001
	Low	216 (27.2)	578 (72.8)		
	Adequate	1470 (35.2)	2710 (64.8)		
**Activity trackers**				54.754	<.001
	Low	175 (22.0)	619 (78.0)		
	Adequate	1486 (35.6)	2694 (64.4)		
**Patient portals**				102.642	<.001
	Low	204 (25.7)	590 (74.3)		
	Adequate	1883 (45.0)	2297 (55.0)		

**Table 3:** Standardized regression coefficients and model analyses for fitness apps.

Model	Predictors	Step 1^a^	*P* value	Step 2^b^	*P* value	*F* (*df*)	*P* value	*R* ^2^	Δ *R*^2^	*P* value
**Ease of use**										
	Age	–.204	<.001	–.205	<.001					
	Sex	.032	.02	.027	.053					
	Work in health care	–.026	.07	–.033	.02					
	Income	.159	<.001	.141	<.001					
	Education	.072	<.001	.058	<.001					
	Asian	–.022	.11	–.013	.34					
	Hispanic	.024	.09	.035	.01					
	African American	.042	.003	.064	<.001					
	Race: other	–.002	.87	–.001	.97	45.937 (9,4894)	<.001	.078		
	NVS^c^ score			.123	<.001	49.255 (10,4893)	<.001	.091	.014	<.001
**Usefulness**										
	Age	–.106	<.001	–.106	<.001					
	Sex	.094	<.001	.092	<.001					
	Work in health care	–.018	.13	–.022	.13					
	Income	.125	<.001	.117	<.001					
	Education	.017	.50	.011	.50					
	Asian	–.005	.95	–.001	.95					
	Hispanic	.029	.02	.035	.02					
	African American	.032	.004	.042	.004					
	Race: other	–.009	.58	–.008	.58	21.214 (9,4892)	<.001	.038		
	NVS score			.056	<.001	20.603 (10,4891)	<.001	.040	.003	<.001

^a^Regression of demographics onto perceived ease of use and perceived usefulness.

^b^Regression of Newest Vital Sign score onto perceived ease of use and perceived usefulness.

^c^NVS: Newest Vital Sign.

**Table 4:** Standardized regression coefficients and model analyses for nutrition apps.

Model	Predictors	Step 1^a^	*P* value	Step 2^b^	*P* value	*F* (*df*)	*P* value	*R* ^2^	Δ *R*^2^	*P* value
**Ease of use**										
	Age	–.145	<.001	–.146	<.001					
	Sex	.085	<.001	.080	<.001					
	Work in health care	–.007	.63	–.015	.28					
	Income	.120	<.001	.100	<.001					
	Education	.094	<.001	.079	<.001					
	Asian	–.027	.06	–.017	.22					
	Hispanic	.021	.15	.033	.02					
	African American	.024	.10	.048	.001					
	Race: other	–.012	.37	–.011	.45	33.261 (9,4875)	<.001	.058		
	NVS^c^ score			.134	<.001	39.002 (10,4874)	<.001	.074	.016	<.001
**Usefulness**										
	Age	–.054	<.001	–.055	<.001					
	Sex	.122	<.001	.119	<.001					
	Work in health care	–.005	.73	–.009	.53					
	Income	.102	<.001	.092	<.001					
	Education	.024	.13	.017	.29					
	Asian	–.022	.13	–.017	.23					
	Hispanic	.045	.002	.050	.001					
	African American	.018	.22	.029	.05					
	Race: other	–.017	.24	–.016	.26	17.479 (9,4874)	<.001	.031		
	NVS score			.063	<.001	17.580 (10,4873)	<.001	.035	.004	<.001

^a^Regression of demographics onto perceived ease of use and perceived usefulness.

^b^Regression of Newest Vital Sign score onto perceived ease of use and perceived usefulness.

^c^NVS: Newest Vital Sign.

**Table 5:** Standardized regression coefficients and model analyses for activity trackers.

Model	Predictor	Step 1^a^	*P* value	Step 2^b^	*P* value	*F* (*df*)	*P* value	*R* ^2^	Δ *R*^2^	*P* value
**Ease of use**										
	Age	–.149	<.001	–.150	<.001					
	Sex	.034	.02	.029	.04					
	Work in health care	–.007	.64	–.015	.29					
	Income	.152	<.001	.132	<.001					
	Education	.094	<.001	.080	<.001					
	Asian	–.026	.07	–.016	.24					
	Hispanic	.020	.16	.032	.03					
	African American	.023	.12	.047	.001					
	Race: other	–.004	.76	–.002	.86	35.460 (9,4883)	<.001	.061		
	NVS^c^ score			.130	<.001	40.54 (10,3882)	<.001	.077	.015	<.001
**Usefulness**										
	Age	–.082	<.001	–.082	<.001					
	Sex	.102	<.001	.100	<.001					
	Work in health care	–.008	.60	–.011	.43					
	Income	.129	<.001	.119	<.001					
	Education	.037	.02	.031	.06					
	Asian	–.003	.90	.002	.91					
	Hispanic	.027	.07	.032	.03					
	African American	.013	.36	.024	.10					
	Race: other	–.009	.51	–.009	.55	20.843 (9,4879)	<.001	.037		
	NVS score			.060	<.001	20.462 (10,4878)	<.001	.040	.003	<.001

^a^Regression of demographics onto perceived ease of use and perceived usefulness.

^b^Regression of Newest Vital Sign score onto perceived ease of use and perceived usefulness.

^c^NVS: Newest Vital Sign.

**Table 6:** Standardized regression coefficients and model analyses for patient portals.

Model	Predictor	Step 1^a^	*P* value	Step 2^b^	*P* value	*F* (*df*)	*P* value	*R* ^2^	Δ *R*^2^	*P* value
**Ease of use**										
	Age	.018	.23	.017	.25					
	Sex	.060	<.001	.056	<.001					
	Work in health care	–.020	.17	–.027	.06					
	Income	.107	<.001	.089	<.001					
	Education	.074	<.001	.062	<.001					
	Asian	–.005	.72	.003	.83					
	Hispanic	.010	.51	.020	.16					
	African American	.028	.60	.049	.001					
	Race: other	–.005	.75	–.003	.83	15.509 (9,4887)	<.001	.028		
	NVS^c^ score			.116	<.001	20.310 (10,4886)	<.001	.040	.012	<.001
**Usefulness**										
	Age	.051	<.001	.050	.001					
	Sex	.106	<.001	.102	<.001					
	Work in health care	–.016	.26	–.022	.13					
	Income	.083	<.001	.070	<.001					
	Education	.040	.01	.031	.052					
	Asian	–.008	.59	–.002	.91					
	Hispanic	.017	.25	.025	.09					
	African American	.038	.01	.053	<.001					
	Race: other	–.039	.01	–.038	.01	14.610 (9,4886)	<.001	.026		
	NVS score			.084	<.001	16.466 (10,4885)	<.001	.033	.006	<.001

^a^Regression of demographics onto perceived ease of use and perceived usefulness.

^b^Regression of Newest Vital Sign score onto perceived ease of use and perceived usefulness.

^c^NVS: Newest Vital Sign.

**Table 7:** Standardized regression coefficients and model analysis for privacy.

Model	Predictors	Step 1^a^	*P* value	Step 2^b^	*P* value	*F* (*df*)	*P* value	*R* ^2^	Δ *R*^2^	*P* value
**Fitness app privacy**										
	Age	–.111	<.001	–.106	<.001					
	Sex	–.093	.001	–.079	.004					
	Work in health care	.005	.86	.006	.83					
	Income	–.044	.16	–.028	.36					
	Education	–.132	<.001	–.115	<.001					
	Asian	.048	.07	.038	.16					
	Hispanic	.024	.38	.011	.69					
	African American	–.007	.80	–.032	.25					
	Race: other	–.048	.08	–.047	.08	8.460 (9,1335)	<.001	.054		
	NVS^c^ score			–.127	<.001	9.776 (10,1334)	<.001	.061	.014	<.001
**Nutrition app privacy**										
	Age	–.092	<.001	–.091	<.001					
	Sex	–.063	.01	–.053	.03					
	Work in health care	–.048	.053	–.040	.11					
	Income	–.076	.01	–.061	.03					
	Education	–.128	<.001	–.118	<.001					
	Asian	.032	.20	.023	.36					
	Hispanic	.013	.61	.007	.84					
	African American	–.003	.90	–.021	.41					
	Race: other	–.048	.05	–.047	.05	9.594 (9,1630)	<.001	.050		
	NVS score			–.097	<.001	10.170 (10,1629)	<.001	.059	.008	<.001
**Activity tracker privacy**										
	Age	–.152	<.001	–.150	<.001					
	Sex	–.060	.02	–.060	.02					
	Work in health care	–.016	.52	–.011	.65					
	Income	–.005	.85	.001	.98					
	Education	–.129	<.001	–.123	<.001					
	Asian	.000	.99	–.001	.98					
	Hispanic	–.002	.94	–.005	.83					
	African American	.003	.91	–.004	.87					
	Race: other	–.028	.26	–.028	.25	8.383 (9,1611)	<.001	.045		
	NVS score			–.049	.053	7.934 (10,1610)	<.001	.047	.002	.053
**Patient portal privacy**										
	Age	–.076	.001	–.075	.001					
	Sex	–.029	.21	–.029	.20					
	Work in health care	–.019	.40	–.021	.36					
	Income	–.008	.74	–.012	.64					
	Education	–.038	.13	–.040	.11					
	Asian	–.009	.68	–.009	.70					
	Hispanic	.007	.75	.010	.67					
	African American	–.045	.05	–.042	.07					
	Race: other	–.052	.02	–.052	.02	2.733 (9,2023)	.004	.012		
	NVS score			.023	.31	2.563 (10,2022)	.004	.013	.001	.31

^a^Regression of demographics onto perceived ease of use and perceived usefulness.

^b^Regression of Newest Vital Sign score onto perceived ease of use and perceived usefulness.

^c^NVS: Newest Vital Sign.

For fitness apps, NVS score was positively associated with both perceived ease of use (*b*=.126, *t*_4892_=8.546, *P*<.001, beta=.123) and usefulness (*b*=.057, *t*_4890_=3.818, *P*<.001, beta=.056) such that as NVS score increased, fitness apps were perceived as easier to use and more useful. Results were similar for NVS score associated with nutrition app ease of use (*b*=.135, *t*_4873_=9.246, *P*<.001, beta=.134) and usefulness (*b*=.063, *t*_4872_=4.236, *P*<.001, beta=.063), activity tracker ease of use (*b*=.133, *t*_4881_=9.005, *P*<.001, beta=.130) and usefulness (*b*=.061, *t*_4877_=4.054, *P*<.001, beta=.060), and patient portal ease of use (*b*=.115, *t*_4885_=7.861, *P*<.001, beta=.116) and usefulness (*b*=.079, *t*_4884_=5.686, *P*<.001, beta=.084).

### Research Question 3

Research question 3 sought to understand how health literacy might influence perceptions of privacy associated with HIT. Hierarchical linear regression analysis suggested that NVS score was significantly associated with perceptions of privacy for fitness apps, nutrition apps, and activity trackers after controlling for demographics (Table 7).

Overall, all four regression models explained a significant proportion of variance in privacy perceptions, ranging from 1.3% to 6.1% (Table 7). NVS score was negatively associated with privacy perceptions of fitness apps (*b*=–.106, *t*_1333_=–4.528, *P*<.001, beta=–.127) and nutrition apps (*b*=–.087, *t*_1628_=–3.825, *P*<.001, beta=–.097). Thus, as NVS score decreased, perceptions of privacy were more likely to be positive. Although the overall models for activity trackers and patient portal privacy were indeed significant, the variance explained was not significantly associated with NVS score in either model (activity trackers: *b*=–.048, *t*_1609_=–1.938, *P*=.053, beta=–.049; patient portal: *b*=.024, *t*_2021_=1.1014, *P*=.03, beta=.023).

### Research Question 4

Research question 4 looked at the association between health literacy and perceptions of trust in various institutions (government, media, technology companies, and health care). Four hierarchical regression models examined the association of NVS score and trust in each institution; the models explained a significant proportion of variance in trust perceptions, ranging from 0.06% to 4.6% (Table 8). After controlling for demographics, NVS score was negatively associated with trust in government (*b*=–.091 *t*_4887_=–5.513, *P*<.001, beta=–.081), media (*b*=–.126, *t*_4880_=–8.494, *P*<.001, beta=–.126), and technology companies (*b*=–.161, *t*_4874_=–10.705, *P*<.001, beta=–.158). However, NVS score was positively associated with trust in health care (*b*=.031, *t*_4868_=2.141, *P*=.03, beta=.032).

**Table 8:** Standardized regression coefficients and model analyses for trust.

Model	Predictors	Step 1^a^	*P* value	Step 2^b^	*P* value	*F* (*df*)	*P* value	*R* ^2^	Δ *R*^2^	*P* value
**Trust in government**										
	Age	–.078	<.001	–.077	<.001					
	Sex	.020	.16	.024	.10					
	Work in health care	–.049	.001	–.044	.002					
	Income	–.034	.03	–.022	.18					
	Education	.009	.56	.018	.27					
	Asian	.050	.001	.044	.002					
	Hispanic	.060	<.001	.052	<.001					
	African American	.103	<.001	.089	<.001					
	Race: other	–.040	.005	–.041	.004	17.518 (9,4889)	<.001	.031		
	NVS^c^ score			–.081	<.001	18.900 (10,4888)	<.001	.037	.006	<.001
**Trust in media**										
	Age	.011	.45	.012	.41					
	Sex	.004	.77	.009	.54					
	Work in health care	–.043	.003	–.035	.02					
	Income	–.006	.71	.013	.41					
	Education	–.035	.03	–.021	.19					
	Asian	.057	<.001	.048	.001					
	Hispanic	.048	.001	.037	.01					
	African American	.077	<.001	.054	<.001					
	Race: other	–.017	.22	–.019	.18	6.966 (9,4882)	<.001	.013		
	NVS score			–.126	<.001	13.576 (10,4881)	<.001	.027	.014	<.001
**Trust in technology companies**										
	Age	–.062	<.001	–.060	<.001					
	Sex	–.019	.20	–.013	.37					
	Work in health care	–.044	.002	–.034	.01					
	Income	–.023	.16	.001	.94					
	Education	–.059	<.001	–.042	.01					
	Asian	.073	<.001	.062	<.001					
	Hispanic	.053	<.001	.038	.02					
	African American	.062	<.001	.034	.02					
	Race: other	–.020	.16	–.022	.12	12.979 (9,4876)	<.001	.023		
	NVS score			–.158	<.001	23.413 (10,4875)	<.001	.046	.022	<.001
**Trust in health care**										
	Age	.028	.06	.028	.06					
	Sex	.034	.02	.033	.02					
	Work in health care	–.040	.02	–.042	.004					
	Income	–.026	.11	–.031	.06					
	Education	.018	.27	.014	.38					
	Asian	–.016	.27	–.014	.34					
	Hispanic	–.015	.31	–.012	.41					
	African American	.011	.47	.017	.27					
	Race: other	–.023	.12	–.022	.12	2.879 (9,4870)	.002	.005		
	NVS score			.032	.03	3.051 (10,4869)	.001	.006	.001	.03

^a^Regression of demographics onto perceived ease of use and perceived usefulness.

^b^Regression of Newest Vital Sign score onto perceived ease of use and perceived usefulness.

^c^NVS: Newest Vital Sign.

## Discussion

The purpose of this study was to better understand how health literacy is associated with HIT adoption, and relevant issues such as information privacy and trust. In this study, patients with low health literacy were less likely to use HIT tools or perceive them as easy or useful, but they perceived information on HIT as private. To our knowledge, this is the first wide-scale investigation of these interrelated concepts.

As might have been expected, HIT adoption—linked to perceived ease of use and perceived usefulness—was associated with higher health literacy. This stands to reason, given that health literacy is defined as how people obtain, understand, use, and communicate about health-related information [1]. Our results suggest that the actual design of HIT apps, ranging from wearable technology to patient portals, has room for improvement so that lower health-literate audiences will perceive the apps as more useful and easy to use. Indeed, Bickmore and Paasche-Orlow [9] argue that researchers do not often consider the limitations of patients of varying abilities when designing HIT tools. Given that more health-literate users still appreciate the simplicity and approach of interventions designed for lower health-literate users [7,35], a focus on design and usability for lower health-literate users would benefit all users. This is particularly true given the importance of first impressions in evaluating technology such as patient portals [36], meaning that gaining attention from patients may be difficult if their initial experiences are not positive. The relationship between health literacy and perceived ease of use was stronger than that between health literacy and perceived usefulness; while users’ perceived usefulness might be driven by some factors beyond the control of HIT developers (eg, potential users might already be successfully managing a chronic condition and see no need for a diet app), perceived ease of use matters for all potential users and a focus on usability could lower barriers to users trying an app and successfully integrating it into their lives.

The association between health literacy and privacy issues related to HIT apps was straightforward: lower health literacy was associated with greater perceptions of privacy when using HIT apps. This relationship points to fruitful directions for future research, including focused study of how users of various health literacy levels make decisions about information to share with apps and by what criteria they judge the privacy protections of various HIT apps. This finding also suggests a need for education on information privacy, perhaps as part of interventions designed to build health literacy and computer self-efficacy skills for underserved populations, to help them make the most informed decisions possible about their health information privacy.

The relationship between health literacy and trust in various stakeholders associated with HIT apps was more nuanced. Less health-literate participants were less trusting of the government, media, and technology companies; the relationship between low health literacy and trust in government as an information source is not new [37], but this research confirms that finding with a more representative sample. At the same time, those with lower health literacy were more likely to place trust in health care providers. Further research is needed to better understand the drivers of these feelings of trust, but they have major implications for how HIT apps might be successfully rolled out to the public. The greater feelings of trust in health care providers among lower health-literate users suggest that companies and government organizations interested in rolling out new HIT to lower health-literate populations should consider partnering with trusted health care providers to help ensure adoption.

This study has several limitations that must be acknowledged when considering the implications of these findings and directions for future research. First and foremost, this was an online survey. While the final sample was generally representative of the US public on key demographic measures, all users must have had some level of comfort with technology to be part of the participant pool—the participants in this study were almost certainly more comfortable with the Internet than were the US public. Additionally, the study sample was more health-literate than the general US population. More targeted data collection focused on less health-literate users is needed to confirm these findings, but the association of health literacy with HIT usage and associated issues in this sample suggests that these associations with less health-literate and technologically sophisticated users may be even more pronounced. Given the recent emergence of HIT, this study only asked participants whether they had ever used the technologies of interest (ie, fitness and nutrition apps, activity trackers, and patient portals); thus, some may have used the HIT only one time while others used it regularly. Future research would benefit from a more precise measure of HIT use. The variety of new HIT apps also means that the potential privacy issues involved in their use is constantly evolving, suggesting more focused attention on measurement of different privacy issues related to HIT usage are needed to strengthen research in this area going forward.

We used a valid and reliable measure of health literacy, the NVS, in this study [26]; however, disagreement exists in the field about the best method for measuring health literacy [38]. Indeed, there are numerous measures of health literacy that capture this concept in a variety of ways, including general and topic-specific health literacies [39]. Future work should explore the impact of general, objective health literacy (as measured in this study) versus self-reported or topic-specific literacy (such as fitness or nutrition health literacy) on HIT use. Finally, while the focus of this study was on the relationship between health literacy level and various factors related to HIT, the proportion of variance explained in each model indicates there may be other important factors that should be considered. Future research should explore patients’ comfort with and previous history of using new technology to find and use health information.

HIT apps, from smartphone apps to wearables devices to patient portals, have seen widespread adoption in recent years. The pace of development and capabilities of such tools will only increase in the future. There is a pressing need to understand how health literacy is related to HIT app adoption and usage to ensure that all users receive the full health benefits from these technological advances, in a manner that protects health information privacy, and that users engage with organizations and providers they trust.

## Abbreviations

EHR: Electronic health record
HIT: Health information technology
HIPAA: Health Insurance Portability and Accountability Act
HITECH: Health Information Technology for Economic and Clinical Health
NVS: Newest Vital Sign
